# Dravet syndrome: a new causative SCN1A mutation?

**DOI:** 10.1002/ccr3.787

**Published:** 2017-03-18

**Authors:** Martin Poryo, Oriana Clasen, Barbara Oehl‐Jaschkowitz, Alexander Christmann, Ludwig Gortner, Sascha Meyer

**Affiliations:** ^1^ Department of Pediatric CardiologySaarland University HospitalHomburg/SaarGermany; ^2^ Department of Pediatrics and NeonatologySaarland University HospitalHomburg/SaarGermany; ^3^ Gemeinschaftspraxis für HumangenetikHomburg/SaarGermany; ^4^ Department of Pediatric NeurologySaarland University HospitalHomburg/SaarGermany

**Keywords:** Channelopathy, Dravet syndrome, SCN1A, severe myoclonic epilepsy of infancy

## Abstract

Dravet syndrome is often caused by SCN1A mutations and has a wide variation in clinical appearance. Indication for genetic analysis should be an epileptic encephalopathy or severe clinical course of seizures in infants with episodes of fever before the first year of life.

## Introduction

The majority of genetic epilepsies with known molecular origin are caused by channelopathies 1. One of the most common channelopathy affects the *α*1 subunit of a neuronal voltage‐gated sodium channel that is coded by the SCN1A gene (Chromosome 2q24.3). Mutations in this gene are observed in over 70% of patients with Dravet syndrome 1, 2, 3, 4, 5, 6, 7.

The great number of newly described mutations in the SCN1A gene demonstrates the importance of this sodium channel for epilepsies 1. In Dravet syndrome, about 95% of the SCN1A mutations occur de novo; the others are familial mutations with a mild phenotype 4, 5. Of note, in SCN1A‐affected children, mutations can be found in the whole gene in contrast to many diseases where the mutations are located at distinct genetic positions 4.

We report on a new SCN1A mutation that to the best of our knowledge has not been described previously.

## Case Report

We report on a 1.4‐year‐old toddler who developed both generalized and unilateral tonic–clonic seizures, myoclonic seizures, as well as seizures with episodes of severe apneic spells with onset of symptoms at the age of 4 months associated with fever following standard vaccination as per STIKO protocol (German vaccination program). Diagnostic work‐up including lumbar puncture, cerebral MRI, and metabolic (amino and organic acids) as well as genetic studies (karyotyping and array CGH) studies were unrevealing. Several EEG recordings (both awake and sleeping) demonstrated fronto‐parietal–temporal epileptic discharges (F8/T8/P8) on the right hemisphere and frontal epileptic discharges (F3/F7) on the left hemisphere as well as occipital slowing.

During the next couple of months, recurrent seizures occurred and antiepileptic treatment was started with oxcarbazepine (maximum dose 42.5 mg/kg body weight) and levetiracetam (maximum dose 19 mg/kg body weight).

The further clinical course was complicated by severe episodes of status epilepticus requiring intensive care treatment and mechanical ventilation.

With regard to the clinical course, Dravet syndrome was suspected and the patient was started on valproic acid (end dose, 42.5 mg/kg; blood concentration: 82.7 mg/L [range: 60–100 mg/L]), while levetiracetam and oxcarbazepine were discontinued. Also, clobazam (end dose, 0.25 mg/kg) and stiripentol (end dose, 19 mg/kg; blood concentration: 16 mg/L [range: 4–22]) were added to the anti‐epileptic drug regimen resulting in full control of seizure activity despite occasional episodes of fever. Moreover, our patient demonstrated normal development with regard to cognition with only very subtle delays in motor skills at the age of 2 years now.

Genetic analysis (Sanger Sequencing) demonstrated a de novo missense SCN1A gene mutation p.Val971Leu with a heterozygous base exchange (c.2911G>C; NM_001165963.1; HGVS) within the coding section (exon 15) of the SCN1A gene. The in silico analysis using the prediction program “Mutation Taster” described this mutation as possibly pathogenic.

Parental genetic analysis was unrevealing.

## Discussion

The severe myoclonic epilepsy in infancy (SMEI) or Dravet syndrome, as it was renamed in 1989, was first described in 1978 by Charlotte Dravet. First manifestation of the disease mostly occurs between the first and eighth months of life, only rarely after the first year of life. Its prevalence is estimated as 1:20.000–40.000, but it might be under‐recognized because of its difficult diagnosis. In about 50% of cases, vaccination precedes the first seizure. These seizures may occur in the context of fever under‐recognized, but also an afebrile first manifestation can be seen in as many as 32–58% of patients. The seizures can be complex partial or generalized tonic, clonic, tonic–clonic or myoclonic – often with a prolonged course. Initial psychomotor development is often normal, but stagnates in the further course, usually in the second year of life. The extent of the psychomotor retardation in the following years can be markedly variable. Treatment of this disease is challenging because of its often poor response to pharmacotherapy 2, 4, 5, 6, 7, 8, 9, 10, 11, 12, 13.

Several antiepileptic drugs such as carbamazepin, lamotrigin, vigabatrin, or phenytoin should be avoided in Dravet syndrome because they can worsen the seizures. Valproic acid, topiramate, stiripentol, ethosuximide, and levetiracetam are effective in the treatment of children with Dravet syndrome. If the response to pharmacotherapy is inadequate, ketogenic diet and vagal nerve stimulator may also be used as alternative treatments 5, 11, 14, 15, 16, 17, 18.

As in our patient, Claes et al. found heterozygous mutation in the SCN1A gene, which could not be found in the corresponding parents. They concluded that the de novo mutations in the SCN1A gene are responsible for Dravet syndrome 2. Also Harkin et al. concluded that the de novo mutation in the SCN1A gene makes a pathogenic impact presumable 4. Alternatively, given the mild delay affecting only motor development in our patient, GEFS+ as a mild phenotypic occurrence of SCN1A mutations may also be considered. Conversely, a positive SCN1A result after a prolonged partial seizure with fever after a vaccination around the sixth month of life is considered proof of the presence of Dravet syndrome 5. Of note, the necessity of intensive anti‐epileptic drug treatment is also suggestive of Dravet syndrome. Furthermore, the computer‐based analysis with the prediction programs PolyPhen2 and MutationTaster classified the mutation in our patient as probably disease causing.

In summary, we present a patient with a Dravet syndrome and a new mutation affecting the SCN1A gene as detailed above. Of note, employing next‐generation sequencing, a number of further genetic alterations were found with hitherto unknown clinical significance. Full seizure control was achieved using an antiepileptic drug regimen including valproic acid, clobazam, and stiripentol. Moreover, so far, a near‐to‐normal development was seen in our patient (now at the age of 2 years).

Our case report contributes to the understanding of underlying genetic alterations and a great variability in phenotypic presentation in children with Dravet syndrome 19, demonstrating an unusual clinical course with a very favorable outcome. Indication to analyze the SCN1A gene should be an epileptic encephalopathy or severe clinical course of seizures in infants with episodes of fever before the first year of life 4, 13.

## Authorship

MP: wrote the first draft, edited the manuscript, and performed the literature search. OC: edited the manuscript and was involved in the literature search. BOJ: made critical review of the manuscript and was involved in the literature search. AC: reviewed the manuscript. LG: edited and critically reviewed the manuscript. SM: edited the manuscript and performed the literature search as well as the critical revision of the same.

## Conflict of Interest

None declared.
